# RETRACTION: MiR‐129‐5p Inhibits Glioma Cell Progression In Vitro and In Vivo by Targeting *TGIF2*

**DOI:** 10.1111/jcmm.70174

**Published:** 2024-11-12

## Abstract

**RETRACTION**: Y. Diao, B. Jin, L. Huang, and W. Zhou, “MiR‐129‐5p Inhibits Glioma Cell Progression In Vitro and In Vivo by Targeting *TGIF2*,” *Journal of Cellular and Molecular Medicine* 22, no. 4 (2018): 2357–2367. doi: 10.1111/jcmm.13529.

The above article, published online on 12 February 2018 in Wiley Online Library (wileyonlinelibrary.com), has been retracted by agreement between the journal Editor‐in‐Chief, Stefan Constantinescu; The Foundation for Cellular and Molecular Medicine; and John Wiley and Sons Ltd. The retraction has been agreed following an investigation into concerns raised by a third party, which revealed inappropriate duplications of image panels (Figure 4A and 6G) between this and other articles that were either previously published or published later in the same year by different group of authors. Thus, the editors consider the conclusions of this manuscript substantially compromised. The authors and their institute were informed of the concerns and the decision to retract but they remained unresponsive.

**RETRACTION**: Y. Diao, B. Jin, L. Huang, and W. Zhou, “MiR‐129‐5p Inhibits Glioma Cell Progression In Vitro and In Vivo by Targeting *TGIF2*,” *Journal of Cellular and Molecular Medicine* 22, no. 4 (2018): 2357–2367. doi: 10.1111/jcmm.13529.

The above article, published online on 12 February 2018 in Wiley Online Library (wileyonlinelibrary.com), has been retracted by agreement between the journal Editor‐in‐Chief, Stefan Constantinescu; The Foundation for Cellular and Molecular Medicine; and John Wiley and Sons Ltd. The retraction has been agreed following an investigation into concerns raised by a third party, which revealed inappropriate duplications of image panels (Figure 4A and 6G) between this and other articles that were either previously published or published later in the same year by different group of authors. Thus, the editors consider the conclusions of this manuscript substantially compromised. The authors and their institute were informed of the concerns and the decision to retract but they remained unresponsive.

